# Metastatic Tumor Burden and Loci as Predictors of First Line Sunitinib Treatment Efficacy in Patients with Renal Cell Carcinoma

**DOI:** 10.1038/s41598-019-44226-y

**Published:** 2019-05-23

**Authors:** Anna M. Czarnecka, Anna Brodziak, Pawel Sobczuk, Cezary Dendek, Dominika Labochka, Jan Korniluk, Ewa Bartnik, Cezary Szczylik

**Affiliations:** 1 Department of Oncology, Military Institute of Medicine, Warsaw, Poland; 2 Present Address: Maria Sklodowska-Curie Memorial Cancer Center and Institute of Oncology, Warsaw, Poland; 3 Department of Experimental and Clinical Physiology, Laboratory of Centre for Preclinical Research, Medical University of Warsaw, Warsaw, Poland; 4 Faculty of Mathematics and Information Science, Warsaw University of Technology, Warsaw, Poland; 5 Institute of Computer Science, Polish Academy of Sciences, Warsaw, Poland; 6 Present Address: Department of Pediatrics and Endocrinology, Medical University of Warsaw, Warsaw, Poland; 7 Institute of Genetics and Biotechnology, Faculty of Biology, University of Warsaw, Warsaw, Poland; 8 Institute of Biochemistry and Biophysics, Polish Academy of Sciences, Warsaw, Poland; 9 Medical Center for Postgraduate Education, Warsaw, Poland; 10 Department of Oncology, European Health Centre, Otwock, Poland

**Keywords:** Renal cell carcinoma, Pathogenesis

## Abstract

The aim of this study was to investigate the prognostic impact of baseline tumor burden and loci on the efficacy of first line renal cancer treatment with sunitinib. Baseline and on-treatment CT scans were evaluated. Both the Kaplan-Meier and Weibull modelling survival estimators have been used to describe sunitinib treatment response. Logistic regression was used to confirm associations between tumor site, burden and survival. Additionally, analysis of the metastases co-occurrence was conducted using the Bayesian inference on treated and external validation cohorts. 100 patients with metastatic clear cell renal cell carcinoma were treated with sunitinib in this study. Presence of metastases in the abdominal region (HR = 3.93), and the number of brain metastases correlate with shorter PFS, while the presence of thoracic metastases (HR = 0.47) with longer PFS. Localization of metastases in the abdominal region significantly impacts risk of metastases development in other locations including bone and brain metastases. Biology of metastases, in particular their localization, requires further molecular and clinical investigation.

## Introduction

Clear cell renal cell carcinoma (ccRCC) is highly resistant to conventional chemotherapy with cytostatic agents. At the same time, metastatic disease is diagnosed in a substantial percentage of ccRCC patients. An estimated 18% of patients with RCC present with metastases at diagnosis (synchronous metastases) and more than 50% develop metastatic RCC after nephrectomy during follow-up (metachronous metastases)^1^. Most recurrences (85%) occur within the first 3 years after nephrectomy or NSS and the mean interval to recurrence is 29.5 months for patients with stage T2 RCC and 22 months for those with stage T3 tumors^2^. Recent advances in understanding of molecular genetics of RCC have enabled the development and implementation of novel targeted therapies (small-molecule targeted kinase inhibitors) and immunotherapies (check-point inhibitors). Sunitinib (SU11248) was identified as a potent inhibitor of vascular endothelial growth factor receptor 2 (VEGFR2) and beta-type platelet-derived growth factor receptor (PDGFRβ) that also inhibits KIT and FLT3 and therefore decreases cancer cell proliferation and survival, and blocks tumor angiogenesis. Sunitinib efficacy was confirmed by two phase II trials and a pivotal phase III trial, as well as in extended access programs. Sunitinib efficacy is defined by objective response (OR) rates between 30% to 50%, and median progression-free survival (PFS) of 11 months in first-line treatment of metastatic RCC (mRCC); or 10.1 months in patients aged ≥65 years; 3.5 months – if patients present with Eastern Cooperative Oncology Group performance status ≥2, 6.0 months – for patients with non-clear cell histology; and 5.3 months – in those with brain metastases; but list of predictive and prognostic factors for that treatment is still not fully defined^3–5^.

Tumor burden (tumor load - TB) is defined as the total amount of tumor (cells/mass) distributed in the patients’ body, including bone marrow. In Response Evaluation Criteria in Solid Tumors (RECIST) analysis TB is considered the sum of the longest diameters of all measurable lesions^6^. For many years, the main modality of mRCC treatment was immunotherapy (interferon-α and/or interleukin-2). Since that time, computed tomography (CT) and magnetic resonance (MR) imaging have been used to assess tumor response based on morphologic (size, location) criteria, specifically by using RECIST. RECIST classification describes lesions’ size and distinguishes 4 types of treatment response – stable disease (SD), partial response (PR), complete response (CR) or progressive disease (PD). The targeted agents – sorafenib, sunitinib, pazopanib, axitinib, cobozantinib or everolimus – act differently than classical cytotoxic chemotherapy agents and most often may induce stabilization of the disease rather than tumor regression. Tyrosine kinase inhibitors (TKIs) including sunitinib inhibit mostly angiogenesis and to lesser extent cancer cell growth (cytostatic effect) and therefore do not rapidly ‘shrink’ tumors as conventional chemotherapy that induces programmed cell death or rapid necrosis (cytotoxicity). The cytostatic effect occurs in G_1_ or G_2_ phase of cell cycle and causes cell cycle arrest. Moreover it must be remembered that cytotoxic properties, as well as cancer stem cell proliferation inhibition, may be partially responsible for the clinical activity of TKIs^7–9^.

With the transformation of mRCC into chronic disease, achieved by the development and clinical implementation of multiple targeted drugs, different profiles of patients can be distinguished. It was observed that some patients progress rapidly during treatment, while others achieve stable disease for a long time^10^. In the current state of knowledge, clinicians still lack the ability to predict to which category each patient will belong (fast, slow, or medium-progressors) but for long time it has been suggested that general tumor and metastatic burden might be one of the factors influencing the outcome^11–13^. Despite many advances in understanding RCC biology^14–17^, there is still a great deal of information to be discovered regarding the behavior of mRCC treated with VEGF-inhibiting agents both in molecular and clinical analyses^18,19^. Overall, measuring the size of tumors remains the simplest way to estimate the severity of disease and possibly predict response/progression on treatment in routine clinical practice world-wide. Despite extensive literature on functional imaging methods^20–22^, only tumor size is globally applied and considered reliable measure in disease assessment.

Our research hypothesis was that prognosis that relies not only on general change of tumor size needs to be supplemented with additional factors/biomarkers to predict patients’ response to treatment. New markers based on additional radiological data of the patient may enable a more accurate prediction of targeted therapies efficacy in patients with mRCC. The aim of the present study was to investigate the possible prognostic role of baseline tumor burden, including metastasis location, metastases co-localization and subsequent local and general tumor shrinkage (depth of remission) in a homogeneous group of patients treated with sunitinib in first line treatment in clinical practice, outside of clinical trials.

## Materials and Patients

### Patients’ characteristics

Patients were treated as previously described^5^. In general, patients who had pathologically confirmed ccRCC, underwent nephrectomy or NSS and developed metastases in the course of the disease were enrolled in the study. Patients in poor performance status and with active brain metastases were excluded from this analysis, since National Health Fund in Poland does not reimburse sunitinib treatment in these patients. Patients previously treated with immunotherapy or other TKIs were also not eligible. Treatment was initiated between 03.2006 and 07.2013 and follow-up was cut off at the end of 09.2015 to enable observation not shorter than expected from median PFS reported in phase III trial^3^. Sunitinib 50 mg was administered orally as first line treatment once a day for 4 weeks, with a following 2-week resting period (4/2 schedule). Sunitinib dose was adjusted to 37.5 or 25 mg according to the type and severity of adverse events. On the baseline, comprehensive review was undertaken. Radiographic images and follow-up images that were obtained after every second cycle of treatment. Baseline screening CT was performed not earlier than 28 days before the start of therapy. Selection of target lesions and response evaluation were performed based on RECIST version 1.1 guidelines. TB was defined as the sum of the longest unidimensional diameter of each target lesion (in cm) and was restricted to axial CT imaging. Data was analyzed independently by 2 researchers. In the case of disagreement between two observers, a consensus was reached after reassessment and consultation with the PIs of the project (AMC, CS). Metastatic lesions treated before sunitinib administration with local modalities i.e. radiotherapy, including stereotactic radiation therapy or ablation, were excluded as target lesions. Change of the sum of diameter (ΔSOD) of the target lesion was documented for all target lesions. Overall survival (OS) definition was described as the time of the start of therapy to death from any cause. PFS definition was described as the time of the start of therapy to date of progression or death from any cause, whichever occurred first. Reduction of tumor size was measured according to percentage change in the sum of the largest diameter of target lesions.

For the purpose of the analysis, metastatic sites were divided into groups: 1 - abdominal, which includes: abdominal lymph nodes, liver, adrenal glands, local recurrence, pancreas, other kidney, spleen, peritoneum and visceral adipose tissue; 2 - thoracic, which includes: lungs, pleura and thoracic lymph nodes; 3 - brain and 4 - bone metastases, which were considered independently. Muscle, abdominal wall, subcutaneous tissue, pelvic lymph nodes and thyroid gland were categorized as ‘other’ metastases (Supplementary Table S1). Patient, tumor, and treatment characteristics were collected via review of the electronic medical record under a pre-defined protocol. This research was approved by the Institutional Ethical Review Board (Agreement No 48/WIM/2014) of The Military Institute of Medicine. Treatment was covered according to the National Health Fund (NFZ) reimbursement regulations (Poland) based on The Agency for Health Technology Assessment and Tariff System (AOTMiT) recommendations. All patients provided informed consent for the treatment.

### Statistical methods

The Accelerated Failure Time parametric model (AFT)^23^ has been used for the purpose of characterizing the OS and PFS with and without presence of the covariates. Based on the published AFT survival modelling results of sunitinib patients^24^ the Weibull distribution has been assumed as a prior underlying failure time model and post-validated^25^. Since the Weibull AFT model is a parametric proportional hazards model the results of the regression are reported in terms of hazard ratios. In order to provide the non-parametric reference for Weibull modelling results, the Kaplan-Meier survival estimators^26^ has also been fitted, with an assessment of the estimation precision in terms of 95% *log-log* confidence intervals^23^, which are also used for calculating the confidence intervals of the median survival estimator. The sensitivity of both Kaplan-Meier and Weibull survival models to inhomogeneity of the patient cohort has been assessed using the bootstrap resampling procedure^27^ (N^2^ = 10,000 repetitions), which provided corrected estimates of median survival and Weibull parameters^28^. Logistic regression analyses were performed on the entire cohort of patients to assess the associations between tumor burden of interest and whether a metastatic site was defined as being significant for treatment duration or/and progression. All statistical calculations were performed using R v.3.3.

### Tumor burden model validation

In the second step of the study we aimed to test the described approach by estimating the values of a nonlinear prognostic factor (depending only on the sites of metastases) and validate its concordance with OS on a validation cohort.

Previously-published data on the correlations between clinical features in the form of prior probability distributions, obtained using the Bayesian inference (Markov chain Monte Carlo – MCMC – sampling), under the distributional assumptions were aggregated. For the purpose of analyzing the impact of the site of metastasis on the patient survival we have selected a corpus of previous studies providing: the frequency of (grouped) RCC metastatic site co-occurrences, conditional baseline distributions of prognostic factors, as well as statistically significant hazard ratios from multivariate PH models. The studies based on the same cohort of patients have been aggregated before the inference step.

The aggregated data concerning the co-occurrence of the metastases in RCC were provided by Bianchi *et al*.^29^. The referenced paper provides the joint distribution of the metastases based on a large sample (N = 11157) of patients. The vast sample of patients allows for the analysis of the metastases co-occurrence and identification of correlation patterns. The sample consists only of RCC patients with metastases, not providing the full-factorial design, and the resulting full log-linear model is rank-deficient. This conditional model however, is well-defined, allows for the typical analysis and is better suited for the problem, lacking the obvious conclusion that presence of metastases is generally correlated. The main group in the tables denotes the metastasis assumed to be present, for the odds ratio to be valid in nested groups. The estimation of the odds ratio and confidence intervals (CIs) presented in the tables is based on the profile likelihood. The presented odds ratios have been preselected using the Holm-Bonferroni method ($\alpha$ = 0.01). The observed frequency is compared to the theoretical case, when metastases are independent. In figures presenting this model one rectangle describes the state of metastases (1 - presence, 0 - absence) in the abdomen, bones, brain or thoracic location. The area of each rectangle is proportional to the observed frequency, and the color denotes its relation to the theoretical case of independence: red meaning “too infrequent to be independent”, and blue - “too frequent to be independent”. The p-value of the hypothesis of the conditional metastases independence is presented below the color scale.

## Results

### General population characteristics

100 patients have been enrolled in the study. The most common metastatic site was the thoracic region. 79 patients developed metastases in this localization. The second most common group of metastases were abdominal (n = 70). Bone metastases were present in 33 patients, while stable brain metastases were diagnosed in 5 patients (Supplementary Table S1). The number of patients with liver metastases occurring through the whole observation period was related to the side of the primary RCC tumor. The difference in the proportion of liver metastases occurrence (right kidney: 43%, left kidney: 23%) has been found statistically significant (p = 0.047) using the 2-sample test for equality of proportions with continuity correction (Supplementary Table S2).

**Figure 1:**
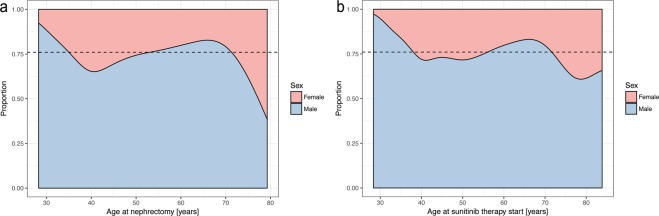
Estimated changes in proportion between sexes vs the age at nephrectomy.

In our study, the primary diagnosis of RCC (followed by nephrectomy providing final histopathological confirmation of ccRCC) occurred mostly in female patients in age groups 40–49 and 50–59 which accounts for almost 60% of all cases of RCC among women. Among male patients RCC diagnosis occurred mostly in the 50–59 age group making up 36.3% of all RCC cases. The overall ratio of male to female patients was approximately 3:1. The male to female ratio was highest in the 39 years and younger group and smallest in the 70 years and older group (Fig. 1, Supplementary Table S3). Older age at nephrectomy was later identified as a negative prognostic factor for PFS (HR = 1.03, p = 0.028) (Supplementary Table S4).

Sunitinib therapy was initiated immediately after the diagnosis of metastatic disease. In our study metastatic disease was most often diagnosed among both male and female patients between 50 and 59 years of age (35.5% of all male RCC and 37.5% in all female patients). The male to female ratio was highest (4.17:1) in the 60–69 age group and lowest in the over 70 years of age group (2.17:1) (Supplementary Tables S3, S5). The effect of the time interval between nephrectomy and sunitinib therapy start on the proportion of sexes was statistically insignificant (p-value = 0.486) (Supplementary Table S6). The time between nephrectomy and start of treatment did not show a statistically significant influence on PFS and OS (p > 0.05) (Supplementary Tables S4, S7).

### Treatment response to sunitinib

**Figure 2:**
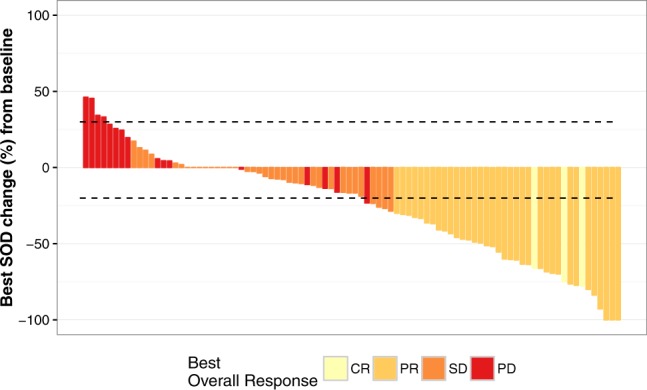
Waterfall plot of the best overall response to sunitinib.

Among 90 patients available for final radiological evaluation 38 (42%) achieved objective responses: 3 CRs and 35 PRs. 36 patients achieved stable disease as their best response (Supplementary Table S8). Overall, 82% of patients experienced some degree of tumor shrinkage (SD + PR + CR) and were considered to achieve clinical benefit (Fig. 2). Approximately 91% of patients had developed PD at the time of data cut-off and analysis. 17 patients developed metastases in different organs at the time of PD, 38 patients had PD as a result of target lesions diameter increase, and 6 patients presented both with new lesions and decrease of target lesions, while all other patients had clinical progression, died or were censored. In our cohort 15 patients developed new metastases in previously uninvolved organs: in bones – 8 patients, in brain – 3, abdominal – 2, thoracic – 1 and other – 1. The most common sites of metastases at treatment initiation were thoracic/lung – 91, abdominal – 89, bones – 49 and brain – 15 and the most common new metastasis organ involved were bones (Supplementary Table S1).

### Progression free survival duration

**Figure 3:**
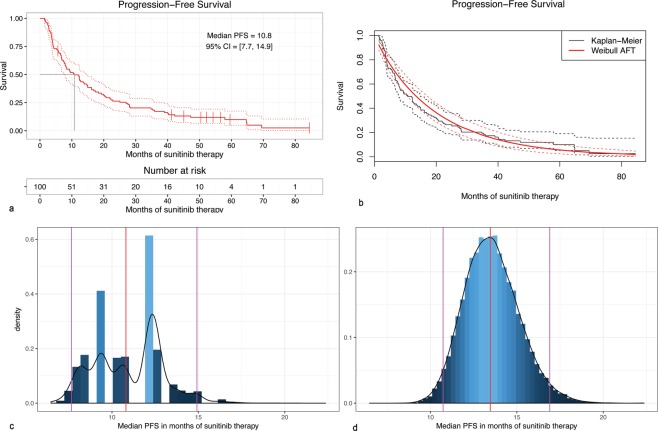
(**a**) Kaplan-Meier estimator of the Progression-Free Survival; (**b**) Comparison of the Kaplan-Meier and Weibull estimates of Progression-Free Survival; (**c**) The density of the bootstrapped Kaplan-Meier median survival estimator; (**d**) The density of the bootstrapped Weibull median survival estimator.

The PFS estimation was based on the 100 observations, and 91 observed events (progressions). The Kaplan-Meier estimated PFS was 10.81 months (95% CI: 7.66–14.92) (Fig. 3a); at the same time the parametric accelerated failure time Weibull survival model provided the estimate of the median PFS of 13.45 months (95% CI: 10.18–16.73). The survival functions estimated by both methods are compared in Fig. 3b. The observed discrepancy of the median PFS value between both survival models may be attributed to the inhomogeneity of the cohort. In order to test this hypothesis, the bootstrap resampling procedure was used to obtain bootstrap estimates (Supplementary Table S9). The density plot of the bootstrap Kaplan-Meier median PFS estimates (Fig. 3c) revealed the bimodal distribution of PFS estimator, stemming from the variability between patients, suggesting the presence of subpopulations of early- and late-progressors. It is worth noticing that the Weibull model captures the central tendency of the sample, favouring the dominating “late-progressors” subpopulation, as the plot of bootstrap Weibull median PFS estimates further showed (Fig. 3c,d).

### Progression free survival risk factors

Univariate analysis of primary tumor size was insignificant for PFS (and OS). The longest dimension of the primary tumor was not found as a prognostic factor of PFS in the univariate Cox PH model (p = 0.22), and was not found to be statistically significant by the Weibull model (p = 0.149). The baseline RECIST ΔSOD was also not prognostic for PFS in the univariate Cox PH model (p = 0.77).

In the next step in the Weibull model several potential predictors of PFS were analyzed (Supplementary Table S4) and identified. Factors such as the presence of metastases in abdominal and ‘other’ groups were shown to have statistically significant negative impact on PFS (HR = 3.93 and HR = 6.88 respectively, p = 0.008 and p = 0.003); however, the presence of metastases in the thoracic group was found to be a positive prognostic factor for PFS (HR = 0.47, p = 0.025). Simultaneously, size of the thoracic metastases has been confirmed as a negative, statistically significant prognostic factor (HR = 1.05 p = 0.001) (Supplementary Table S4). The number of brain metastases was found to be a negative statistically significant prognostic factor of PFS (HR = 3.83, p = 0.021). The total size of nodal metastases, old age at the time of nephrectomy, the size of the primary tumor, involvement of both: measurable and nonmeasurable abdominal lymph nodes have been rejected as prognostic factors of PFS (p > 0.05) (Supplementary Table S4).

### Overall survival duration

**Figure 4:**
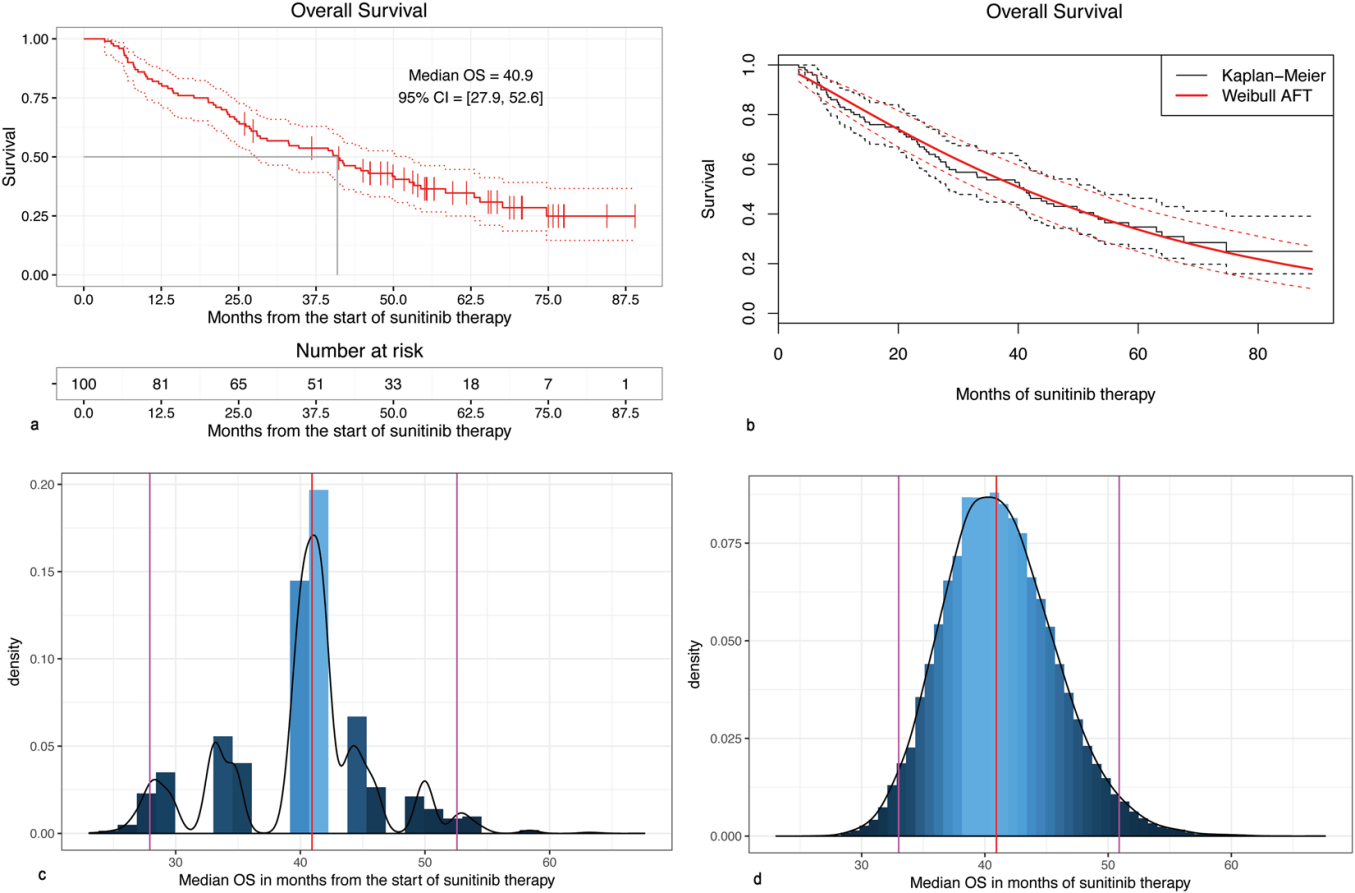
(**a**) Kaplan-Meier estimator of the Overall Survival; (**b**) Comparison of the Kaplan-Meier and Weibull estimates of Overall Survival; (**c**) The density of the bootstrapped Kaplan-Meier median OS estimator; (**d**) The density of the bootstrapped Weibull median OS estimator.

The OS estimation was based on the 100 observations, and 66 observed final events (deaths). The Kaplan-Meier estimator of the OS provided the estimate of the median OS as 40.94 months (95% CI: 27.93–52.57) (Fig. 4a), while the Weibull survival model provides the estimate of 40.87 months (95% CI: 32.34–49.40). As in the case of PFS the results were validated using bootstrap resampling (Fig. 4c,d). The Weibull model closely matched the Kaplan-Meier median OS (Supplementary Table S10, Fig. 4b).

### Overall survival risk factors

A significant relationship was found between the absolute number of metastatic sites and OS (p < 0.05). At the same time the baseline RECIST ΔSOD has been rejected as a prognostic factor of OS in the univariate Cox PH model (p = 0.17), and in the Weibull model (p = 0.15). The sum of sizes of both: brain and thoracic metastases were identified as negative, statistically significant factors of OS (HR = 1.07, HR = 1.03 and p = 0.004, p = 0.019 respectively) (Supplementary Table S7). At the same time, the total size of metastases located in lymph nodes was found to have a statistically significant negative impact on PFS (HR = 1.02 p = 0.023). Neither the presence of metastases in the abdominal group, nor the longest dimension of the primary tumor has a statistically significant influence on OS (p > 0.05). Also, coefficients such as: bone metastases, time between nephrectomy and treatment start or the size of extranodal metastases greater than 8.0 cm failed to reach statistical significance (p > 0.05) for OS impact.

### Metastasis location burden

The basic multivariate Cox PH model fitted using location-grouped metastases as predictors has failed to reach statistical significance for abdominal and other groups, leaving in the resulting model only bone, thoracic and brain as predictors, and resulting in the Harrell concordance index = 0.547. The Bayesian-estimated metastasis prognostic factors – for each patient being a single number - allowed for inclusion of information concerning all the metastasis groups, and resulted in statistically significantly better univariate Cox PH fit (C-index = 0.583) (Supplementary Table S11).

**Figure 5:**
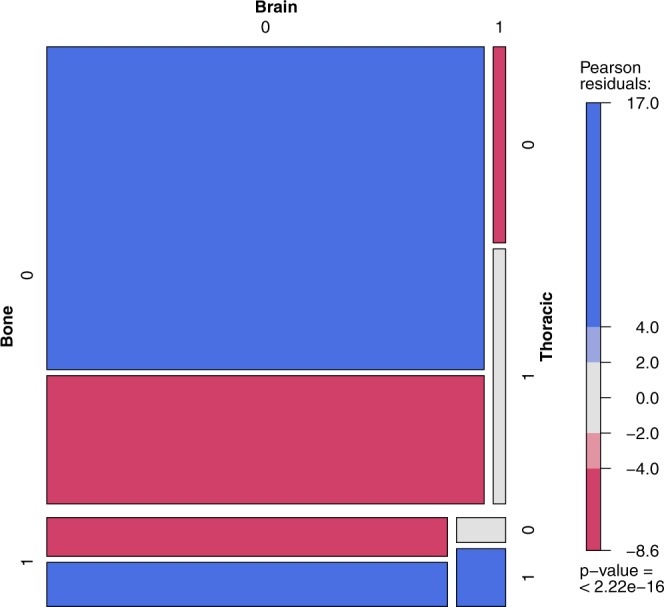
The mosaic plot of metastases co-occurrence frequency among the patients with abdominal metastases.

**Figure 6:**
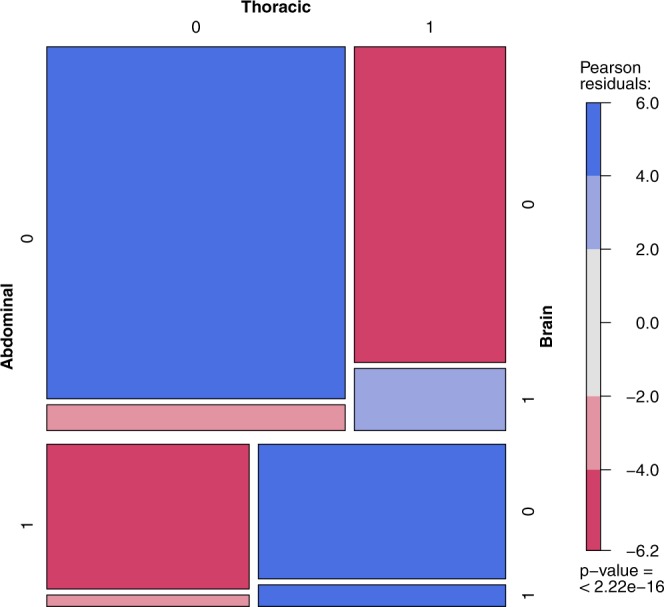
The mosaic plot of metastases co-occurrence frequency among the patients with bone metastases.

**Figure 7:**
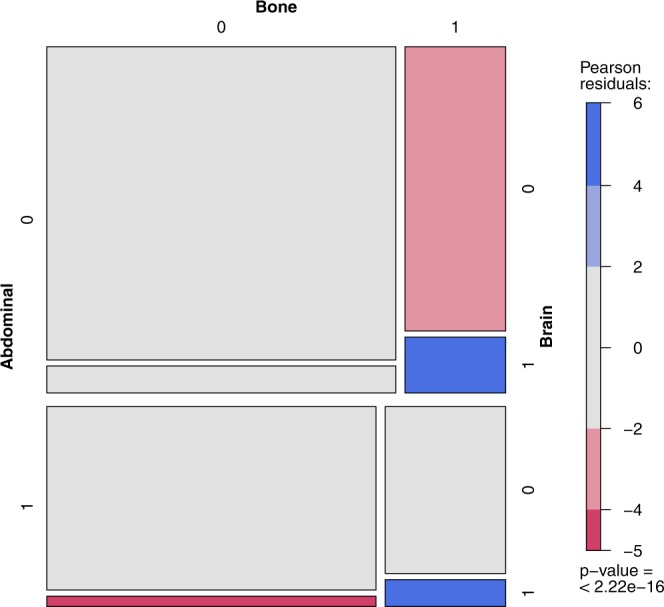
The mosaic plot of metastases co-occurrence frequency among the patients with thoracic metastases.

**Figure 8:**
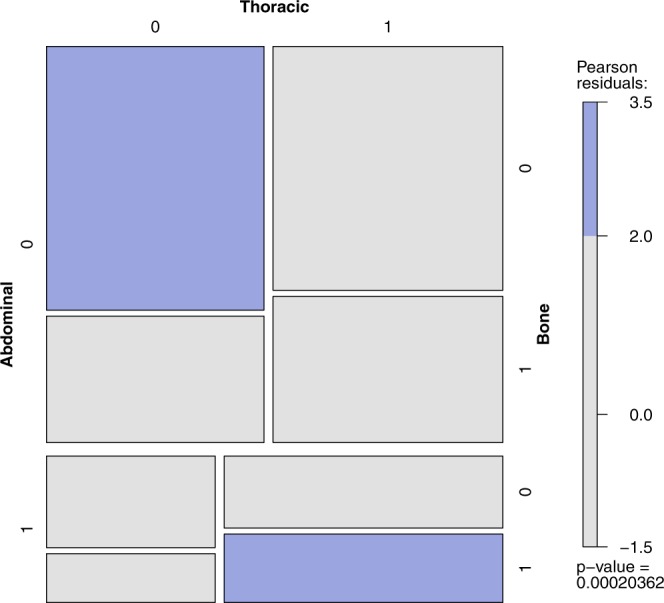
The mosaic plot of metastases co-occurrence frequency among the patients with brain metastases.

Basic metastasis co-localization model (Supplementary Tables 12–15) defined significance as α ≤ 0.01. The most common primary metastases location found was abdominal (Supplementary Table S1) also in patients with co-occurrence of other metastases (Supplementary Table S12). Localization of metastases in the abdominal region statistically and clinically significantly impacted the risk of metastases development/diagnosis in another location including bone vs brain – with 4.31 odds ratio, brain vs thoracic – 3.48 and bone vs thoracic – 2.96 (Fig. 5). Less significant effects were reported for co-occurrence of lesions with bone metastases, including brain vs thoracic: – 2.44 (Fig. 6, Supplementary Table S13) or for thoracic metastases – with bone vs brain – 2.41 (Fig. 7, Supplementary Table S14). The highest odds ratio was found for co-occurrence of metastases when brain metastases are found (Fig. 8, Supplementary Table S15). The odds ratios (Supplementary Table S11) suggested the strong effect of the metastases co-occurrence, that may be explained by the tumor spread using similar or co-activated metastatic invasion mechanisms, as confirmed by external validation (Supplementary Tables S12–15, Figs 5–8).

## Discussion

The findings of this analysis provide insight into how TB and its characteristics, i.e. metastases location, influence outcomes of ccRCC patients treated with sunitinib. Although in our cohort progression of existing sites accounted for the majority of cases of PD, at the same time new organ sites, mostly bones and brain, represented a significant cause of PD. Our analysis of total TB impact on sunitinib efficacy in RCC patients is in accordance with data available for everolimus from RECORD-3 trial, in which the baseline sum of longest tumor diameters was reported as a predictive factor of OS^30^. Likewise, in a mixed population of 85 patients from the sorafenib arm of phase III TARGET trial and 39 patients from phase II sunitinib continuous dose trial, TB was indicated as possible independent prognostic and predictive marker. TB was shown to be significantly associated with prognosis independently of localization of metastases and other clinical factors^31^. In particular, it was previously confirmed in the retrospective analysis of 69 patients treated with sunitinib, that lower total TB, including lower TB above the diaphragm, as well as fewer metastatic sites, are significantly associated with longer OS and slower growing disease^32^. Also baseline TB at the time of second-line therapy is a predictor of OS (but not second line PFS). Another report of 68 patients noted median second-line TB of 57.7 cm^12^. The patients with high TB had significantly shorter PFS and OS, compared to those with low TB – 4.36 vs. 8.19 months of PFS and 9.6 vs. 23.5 months of OS respectively^12^. In our cohort, the total size of metastases located in lymph nodes was found to have a statistically significant negative impact on PFS in first line treatment. In general, higher tumor growth rate at first cycle was associated with worse PFS (HR 3.61) and shorter OS (HR 4.69). Tumor growth rate was an independent prognostic factor, also independent of the Motzer score and of the treatment arm in phase III TARGET trial^33^.

Later reports have shown that a sunitinib-induced decrease in TB is associated with longer PFS, OS and reduced risk of death^32^. Tumor shrinkage defined as ≥10% decrease in sum of the longest diameter (−10%SLD) correlated with time-to-treatment failure (TTF) and OS^34^. More than −10%SLD significantly differentiated potential responders from non-responders with median TTF 8.4 and 4.1 months respectively. Partial response as defined by RECIST was not characteristic for rapid responders. The −10%SLD was also significantly predictive of longer median OS of 35.1 months in responders. Receiver-operating characteristics analysis curve analysis revealed that −9.3% in SLD is the optimal threshold to distinguish responders from non-responders^34^. Moreover, deep tumor shrinkage of at least 60% that is reported to occur in 10% of patients is associated with median OS of 54.5 months^35^.

Before this report, more studies investigated the impact of metastasis location on patients’ prognoses (Supplementary Table S16). Patients with lung and adrenal metastases are known to have 5-year OS rates of around 40 and 60% for single site metastasis, while those with bone and liver - only 15%^36^ Particularly interesting analysis of 34 long term responders who achieved CR (9%), PR (70%) or SD (21%) for at least 18 months on sunitinib treatment confirmed that lack of bone or lung metastasis is predictive of long-term response^37^. This correlation was supplemented with a complementary hypothesis that greater general overall tumor control with TKIs enables metastases development in organ sites like bone or brain, that would otherwise not have been observed due to shorter survival without treatment^32^. This hypothesis can be explained by an observation of restricted transport of sorafenib and sunitinib across the blood-brain barrier. Median time to development of brain metastases is 28 months, but only 11.5 months without therapy^38^. Furthermore, the distribution of metastatic sites was found to be different between early and late responders to sunitinib treatment in a large analysis of 1059 patients treated in six clinical trials. Early responders were carrying metastases in lungs in 84% and in bones in 27%, but late responders in lung in 70% (p = 0.002), and in bones in 19% (p = 0.055)^10^.

Metastases in the lungs, bones and liver were known to be associated with significantly shorter OS on pazopanib, sorafenib, and sunitinib^13^. Our results confirm the abovementioned trend, as we identified sum brain and thoracic metastases as negative, statistically significant factors of OS. On the contrary in our cohort of patients neither the presence of metastases in the abdominal group, nor the longest dimension of the primary tumor show statistically significant influence on OS (all p > 0.05). Previously, the rate of liver metastases was reported as high as 9.3 to 18% in the RCC population. The prognosis of RCC patients with liver metastases is poor, with median OS between 7.6 and 12 months. The presence of liver metastases is therefore correlated with shorter OS compared to OS of patients with metastases in other locations i.e. lung of lymph nodes^39^.

In contrast to patients with liver metastases, patients with pancreatic metastases are known to have indolent disease and longer median OS. Reported median OS for patients with pancreatic metastases is 39–46.1 months, while for patients without pancreatic metastases it is 23.1–26 months, but with all other secondary tumor locations^40–42^. It has even been proposed that pancreatic location of metastases is characteristic for RCC tumors with a less aggressive phenotype. We have previously shown that the presence of pancreatic metastases was not an independent prognostic factor in multivariate analysis, but it was an indicator of an indolent course of the disease in the general RCC population^42^.

Different responses to TKI treatment of particular metastases may be explained not only by the size of particular metastases, but also by location related blood perfusion, differential tumor niche^43^, and genetic background of nodules^44^. As a consequence, different metastatic sites may have a different biology and different response to VEGF inhibition and different TKI exposure and, as a result, may affect clinical outcome^32^.

We believe that selected clinical variables, including TB and TB pattern analysis, can help physicians to make treatment decisions in the future^45^ and individual patients could be scored with radiological parameters described within this project. In the analysed RCC patients (Supplementary Table S1), and large validation cohort (Supplementary Tables S12–15), the most common metastases were found in the abdominal and thoracic regions both as single group of metastases, as well as in patients with other location of metastases co-occurrence. Presence of metastases in the abdominal region significantly modifies the risk of developing other metastases and the course of the disease. Abdominal metastases change rate the proportion of metastases in bones vs brain – with 4.31 odds ratio, in brain vs thoracic – 3.48, and in bones vs thoracic – 2.96 (Fig. 5). A less significant effect is exerted by bone metastases influencing the ratio of brain vs thoracic metastases (Fig. 8, Supplementary Table S13) and by thoracic metastases – influencing the development of bone and brain metastases (Fig. 6, Supplementary Table S14). The highest risk of multiple metastases location was found for co-occurrence with brain metastases (Fig. 7, Supplementary Table S15). Such co-development of metastases may arise as result of tumor spread using similar or co-activated metastatic invasion mechanisms. Altogether the current results of TB and the characteristic impact on the efficacy of ccRCC sunitinib treatment require further evaluation in large prospective trials in order to develop prognostic and predictive scales or nomograms^46^ and integrate these tools into routine clinical practice and influence management of patients with metastatic ccRCC. Cross-sectional radiographic imaging in these patients may be used not only in tumor staging but also as a prognostic tool.

The biology of the particular sites of metastases requires further investigation. The underlying molecular mechanisms of specific organotropism toward selected secondary sites remains poorly understood in RCC and other cancers. Whole genome studies, microarray or next generation sequencing analysis, as well as fluorescent and bioluminescent imaging, may reveal how RCC subtypes differ not only in primary tumor characteristics but also in their metastatic behavior, in particular lung- or abdomen-homing characteristics or bone-seeking phenotype. Understanding the physiological link between the deregulation of specific genes and homing of certain metastases may impact treatment of RCC patients in the future^17,43^.

### Ethical conduct of research

The institutional ethics committee approved the study (Agreement No 48/WIM/2014).

## Supplementary information

Supplementary Information
