# Supplementary material for: Metastatic Tumor Burden and Loci as Predictors of First Line Sunitinib Treatment Efficacy in Patients with Renal Cell Carcinoma
Source: Sci Rep. 2019 May 23;9:7754. doi: 10.1038/s41598-019-44226-y (PMC6533291; doi:10.1038/s41598-019-44226-y)
Supplement: Supplementary file 1 — Supplementary Information [file 41598_2019_44226_MOESM1_ESM.docx]

# METASTATIC TUMOR BURDEN AND LOCI AS PREDICTORS OF FIRST LINE SUNITINIB TREATMENT EFFICACY IN PATIENTS WITH RENAL CELL CARCINOMA

Anna M. Czarnecka MD PhD^1^*, Anna Brodziak MD^1,2,3^, Pawel Sobczuk MD^1,2,3^, Cezary Dendek MSc^4,5^, Dominika Labochka MD^1,6^, Jan Korniluk MD PhD^1^, Ewa Bartnik Prof. PhD^7,8^, Cezary Szczylik Prof. MD PhD^1,9.10^

1 – Department of Oncology, Military Institute of Medicine, Warsaw, Poland

2 – current address: Maria Sklodowska-Curie Memorial Cancer Center and Institute of Oncology, Warsaw, Poland.

3 – Department of Experimental and Clinical Physiology, Laboratory of Centre for Preclinical Research, Medical University of Warsaw, Warsaw, Poland

4 – Faculty of Mathematics and Information Science, Warsaw University of Technology, Warsaw, Poland

5 – Institute of Computer Science, Polish Academy of Sciences, Warsaw, Poland

6 – current address: Department of Pediatrics and Endocrinology, Medical University of Warsaw, Warsaw, Poland

7 – Institute of Genetics and Biotechnology, Faculty of Biology, University of Warsaw, Warsaw, Poland.

8 – Institute of Biochemistry and Biophysics, Polish Academy of Sciences, Warsaw, Poland.

9 – current address: Medical Center for Postgraduate Education , Warsaw, Poland

10 – current address: Department of Oncology, European Health Centre, Otwock, Poland.

# Supplementary Tables

Table S1. Anatomical sites of metastases used in radiological analysis

| **Group** | **Organ** | **Number of patients (total n=100)** |
| --- | --- | --- |
| **Thoracic** | | **79** |
|  | Lungs | 78 |
|  | Thoracic lymph nodes | 57 |
|  | Pleura | 2 |
| **Abdominal** | | **70** |
|  | Abdominal and pelvic lymph nodes | 41 |
|  | Liver | 28 |
|  | Contralateral adrenal gland | 21 |
|  | Local reccurence | 23 |
|  | Pancreas | 14 |
|  | Contralateral kidney | 14 |
|  | Ipsilateral adrenal gland | 4 |
|  | Peritoneum and visceral adipose tissue | 4 |
| **Brain** | | **5** |
| **Bones** | | **33** |
| **Other** | | **10** |
|  | Muscle | 7 |
|  | Abdominal wall | 4 |
|  | Subcutaneous tissue | 1 |
|  | Thyroid gland | 1 |

table S2. Primary tumor location (side) vs liver metaSTASES

| Left kidney | Right kidney | n | Liver metastases |
| --- | --- | --- | --- |
| - | + | 47 | 20 (42.55%) |
| + | - | 39 | 9 (23.08%) |
| + | + | 2 | 1 (50%) |
| unknown | unknown | 12 | 7 (58.33%) |

Table S3. Sex proportion in DIFFERENT AGE groups

|  | Age at nephrectomy | | Age at therapy start | |
| --- | --- | --- | --- | --- |
| Age group | Female | Male | Female | Male |
| 39 or less | 0 | 4 (5.63%) | 0 | 3 (3.95%) |
| 40 – 49 | 7 (29.2%) | 12 (16.9%) | 3 (12.5%) | 8 (10.5%) |
| 50 – 59 | 7 (29.2%) | 26 (36.6%) | 9 (37.5%) | 27 (35.5%) |
| 60 – 69 | 4 (16.7%) | 23 (32.4%) | 6 (25%) | 25 (32.9%) |
| 70 or more | 6 (25%) | 6 (8.45%) | 6 (25%) | 13 (17.1%) |
| NA | 0 | 5 | - | - |

Table S4. PFS – STAGEWISE Coefficients of the Progression-Free Survival Weibull model For factorial predictors

| **Predictor** | **Level** | **N** | **HR** | **95% CI** | **p-value** |
| --- | --- | --- | --- | --- | --- |
| Sum of Diameters of thoracic metastases [mm] |  |  | 1.05 | (1.03 – 1.07) | 0.001 |
| Metastases in group Other | absent | 87 | 6.88 | (1.85 – 25.55) | 0.003 |
|  | present | 13 |  |  |  |
| Metastases in group Abdominal | absent | 30 | 3.93 | (1.40 – 11.06) | 0.008 |
|  | present | 70 |  |  |  |
| Non-measurable metastases only | no | 90 | 3.18 | (1.22 – 8.28) | 0.016 |
|  | yes | 10 |  |  |  |
| Sum of Diameters of other metastases [mm] |  |  | 0.96 | (0.92 – 0.99) | 0.016 |
| Number of brain metastases | 0 | 95 | 3.83 | (1.18 – 12.39) | 0.021 |
|  | 1+ | 5 |  |  |  |
| Metastases in group Thoracic | absent | 21 | 0.47 | (0.24 – 0.93) | 0.025 |
|  | present | 79 |  |  |  |
| Abdominal lymph nodes involvment (measurable) | absent | 87 | 2.11 | (0.94 – 4.75) | 0.067 |
|  | present | 13 |  |  |  |
| Lymph nodes - Total Tumor Burden [mm] |  |  | 0.99 | (0.97 – 1.00) | 0.084 |
| Age at nephrectomy [years] |  |  | 1.02 | (1.00 – 1.04) | 0.096 |
| Measurable extranodal abdominal metastases | absent | 46 | 0.46 | (0.19 – 1.15) | 0.096 |
|  | present | 54 |  |  |  |
| Number of non-measurable abdominal metastases | 0 | 87 | 0.52 | (0.22 – 1.24) | 0.136 |
|  | 1+ | 13 |  |  |  |
| Abdominal lymph nodes involvment (non-measurable) | absent | 72 | 0.59 | (0.29 – 1.19) | 0.139 |
|  | present | 28 |  |  |  |
| Longest dimension of primary tumor [mm] |  |  | 1.00 | (1.00 – 1.01) | 0.149 |

Table S5. Frequency OF Metastatic RENAL Cell carsinoma IN young and elderly patients based on the definition of elderly patients (65+ vs 70+ years of age)

|  |  | **65+** | **70+** | **p** |
| --- | --- | --- | --- | --- |
| **Male** | young | 49 | 63 |  |
|  | elderly | 27 | 13 | 0.016 |
| **Female** | young | 16 | 18 |  |
|  | elderly | 8 | 6 | 0.752 |
| **Overall** | young | 65 | 81 |  |
|  | elderly | 35 | 19 | 0.011 |

Table S6. Time between nephrectomy and sunitinib therapy start

| **Months** | **Female** | **Male** |
| --- | --- | --- |
| **0 – 2** | 3 (12%) | 13 (18%) |
| **2 – 4** | 2 (8.3%) | 14 (20%) |
| **4 - 11** | 4 (17%) | 11 (15%) |
| **11 - 40** | 6 (25%) | 10 (14%) |
| **40 - 70** | 6 (25%) | 10 (14%) |
| **70+** | 3 (12%) | 13 (18%) |
| **NA** | 0 | 5 |

Table S7. OS - STAGEWISE Coefficients of the Overall Survival Weibull model For factorial predictors

| Predictor | Level | N | HR | 95% CI | p-value |
| --- | --- | --- | --- | --- | --- |
| Measurable extranodal abdominal metastases | absent | 46 | 0.18 | (0.07 – 0.52) | 0.001 |
|  | present | 54 |  |  |  |
| Sum of Diameters of brain metastases [mm] |  |  | 1.07 | (1.02 – 1.12) | 0.004 |
| Sum of Diameters of thoracic metastases [mm] |  |  | 1.03 | (1.01 – 1.06) | 0.019 |
| Lymph nodes - Total Tumor Burden [mm] |  |  | 1.02 | (1.00 – 1.04) | 0.023 |
| Abdominal lymph nodes involvment (non-measurable) | absent | 72 | 0.40 | (0.16 – 0.97) | 0.038 |
|  | present | 28 |  |  |  |
| Measurable extranodal metastases in group ,,other” | absent | 90 | 2.62 | (0.99 – 6.96) | 0.051 |
|  | present | 10 |  |  |  |
| Metastases in group Abdominal | absent | 30 | 2.59 | (0.93 – 7.26) | 0.063 |
|  | present | 70 |  |  |  |
| Thoracic lymph nodes involvment (non-measurable) | absent | 77 | 2.01 | (0.95 – 4.23) | 0.065 |
|  | present | 23 |  |  |  |
| Bone metastases | absent | 67 | 1.73 | (0.95 – 3.16) | 0.074 |
|  | present | 33 |  |  |  |
| Time between nephrectomy and treatment start [years] |  |  | 0.35 | (0.10 – 1.16) | 0.083 |
| Measurable extranodal thoracic metastases | absent | 54 | 0.41 | (0.13 – 1.29) | 0.119 |
|  | present | 46 |  |  |  |
| Abdominal lymph nodes involvment (measurable) | absent | 87 | 2.05 | (0.80 – 5.26) | 0.137 |
|  | present | 13 |  |  |  |
| Extranodal Total Tumor Burden > 80 mm | less than 80 mm | 72 | 1.87 | (0.81 – 4.33) | 0.144 |
|  | 80 mm or more | 28 |  |  |  |
| Longest dimension of primary tumor [mm] |  |  | 1.00 | (0.99 – 1.00) | 0.337 |

Table S8. Best Overall Response among patients

| Best Overall Response | Number of patients |
| --- | --- |
| CR | 3 |
| PR | 35 |
| SD | 36 |
| PD | 16 |
| NN/NE | 10 |

Table S9. Estimation of median Progression-Free Survival

| Estimation method | median | 95% CI (lower) | 95% CI (upper) |
| --- | --- | --- | --- |
| Kaplan-Meier | 10.809 | 7.655 | 14.916 |
| Bootstrapped Kaplan-Meier | 10.809 | 7.918 | 14.916 |
| Weibull model | 13.454 | 10.182 | 16.726 |
| Bootstrapped Weibull model | 13.453 | 10.737 | 16.927 |

Table S10. Estimation of median Overall Survival

| Estimation method | median | 95% CI (lower) | 95% CI (upper) |
| --- | --- | --- | --- |
| Kaplan-Meier | 40.936 | 27.926 | 52.567 |
| Bootstrapped Kaplan-Meier | 40.936 | 27.926 | 52.567 |
| Weibull model | 40.869 | 32.341 | 49.398 |
| Bootstrapped Weibull model | 40.968 | 33.144 | 50.911 |

Table S11. The pairwise metastases co-dependency when the main metastaseS ARE present. The dependency is measured by the odds ratio. All OF the results are statistically significant.

| Main group | Nested groups (1) | Nested groups (2) | Odds ratio | 95% CI (lower) | 95% CI (upper) |
| --- | --- | --- | --- | --- | --- |
| Thoracic | Bone | Abdominal | 1.268 | 1.116 | 1.441 |
| Bone | Thoracic | Abdominal | 2.409 | 2.068 | 2.809 |
| Thoracic | Bone | Brain | 2.411 | 1.989 | 2.920 |
| Bone | Brain | Thoracic | 2.439 | 1.940 | 3.074 |
| Abdominal | Bone | Thoracic | 2.960 | 2.573 | 3.407 |
| Abdominal | Brain | Thoracic | 3.481 | 2.677 | 4.550 |
| Abdominal | Bone | Brain | 4.311 | 3.308 | 5.604 |

Table S12. The frequency table of metastases sites co-occurence among the patients with abdominal metastases

| Thoracic | Brain | Bone | N (observed) | N (independent) | Fraction (observed) | Fraction (independent) |
| --- | --- | --- | --- | --- | --- | --- |
| 0 | 0 | 0 | 3474 | 3188 | 58.25 | 53.46 |
| 0 | 0 | 1 | 405 | 620 | 6.79 | 10.40 |
| 0 | 1 | 0 | 60 | 136 | 1.01 | 2.28 |
| 0 | 1 | 1 | 32 | 26 | 0.54 | 0.44 |
| 1 | 0 | 0 | 1381 | 1600 | 23.16 | 26.83 |
| 1 | 0 | 1 | 460 | 311 | 7.71 | 5.22 |
| 1 | 1 | 0 | 78 | 68 | 1.31 | 1.14 |
| 1 | 1 | 1 | 74 | 13 | 1.24 | 0.22 |

TABLE S13. The frequency table of metastases sites co-occurence among the patients with bone metastaseS

| Brain | Thoracic | Abdominal | N (observed) | N (independent) | Fraction (observed) | Fraction (independent) |
| --- | --- | --- | --- | --- | --- | --- |
| 0 | 0 | 0 | 1420 | 1236 | 43.45 | 37.83 |
| 0 | 0 | 1 | 405 | 523 | 12.39 | 15.99 |
| 0 | 1 | 0 | 646 | 824 | 19.77 | 25.21 |
| 0 | 1 | 1 | 460 | 348 | 14.08 | 10.66 |
| 1 | 0 | 0 | 104 | 142 | 3.18 | 4.35 |
| 1 | 0 | 1 | 32 | 60 | 0.98 | 1.84 |
| 1 | 1 | 0 | 127 | 95 | 3.89 | 2.90 |
| 1 | 1 | 1 | 74 | 40 | 2.26 | 1.23 |

**Table S14. THE FREQUENCY TABLE OF METASTASES SITES CO-OCCURENCE AMONG THE PATIENTS WITH THORACIC AREA**

| Brain | Bone | Abdominal | N (observed) | N (independent) | Fraction (observed) | Fraction (independent) |
| --- | --- | --- | --- | --- | --- | --- |
| 0 | 0 | 0 | 2467 | 2387 | 45.31 | 43.84 |
| 0 | 0 | 1 | 1381 | 1378 | 25.36 | 25.31 |
| 0 | 1 | 0 | 646 | 754 | 11.86 | 13.85 |
| 0 | 1 | 1 | 460 | 435 | 8.45 | 7.99 |
| 1 | 0 | 0 | 212 | 237 | 3.89 | 4.34 |
| 1 | 0 | 1 | 78 | 137 | 1.43 | 2.51 |
| 1 | 1 | 0 | 127 | 75 | 2.33 | 1.37 |
| 1 | 1 | 1 | 74 | 43 | 1.36 | 0.79 |

**TABLE S15. THE FREQUENCY TABLE OF METASTASES SITES CO-OCCURENCE AMONG THE PATIENTS WITH BRAIN METASTASES**

| Bone | Thoracic | Abdominal | N (observed) | N (independent) | Fraction (observed) | Fraction (independent) |
| --- | --- | --- | --- | --- | --- | --- |
| 0 | 0 | 0 | 217 | 189 | 24.00 | 20.92 |
| 0 | 0 | 1 | 60 | 70 | 6.64 | 7.73 |
| 0 | 1 | 0 | 212 | 225 | 23.45 | 24.87 |
| 0 | 1 | 1 | 78 | 83 | 8.63 | 9.19 |
| 1 | 0 | 0 | 104 | 112 | 11.50 | 12.43 |
| 1 | 0 | 1 | 32 | 42 | 3.54 | 4.60 |
| 1 | 1 | 0 | 127 | 134 | 14.05 | 14.78 |
| 1 | 1 | 1 | 74 | 49 | 8.19 | 5.47 |

**TABLE S16. PROGNOSTIC FACTORS ASSOCIATED WITH SUNITINIB RESPONSE IN PATIENTS WITH METASTATIC RENAL CELL CARCINOMA IN MULTIVARIATE ANALYSES**

| Factor | Significant for PFS  (months) | Significant for OS  (months) | Total no of pts | Prior cytokine treatment | ccRCC only (yes, no; % of non-ccRCC) | II line treatment after sunitinib  (drug) | % of patients treated with sunitinib as 1^st^ line TKI | % of patients treated with sunitinib as 2^nd^ line TKI | Patients treated with other TKI as 1^st^ line drug % | Reference |
| --- | --- | --- | --- | --- | --- | --- | --- | --- | --- | --- |
| Total lesion glycolysis (high TLG group) | ND | 45.4 | 44 | ND | No, 25% | ND | 66% | 0% | SOR 11%, EVE 11%, TEM 9%, PAZ 2% | (Yoon, Paeng et al. 2013) |
| TB (TB above median vs TB below median) | 4.2 vs 5.6 | 16.4 vs 27.4 | 124 | 100% | yes | ND | 31% | 0% | 35% SOR, 34% BSC | (Iacovelli, Lanoy et al. 2012) |
| TB (1.3-9.4 vs 9.5-19 cm vs 19.1-47.3 cm) | 5.6 vs 6.6 vs 4.2 | 42.1 vs 20.1 vs 11.2 | 124 | 100% | yes | ND | 31% | 0% | 35% SOR, 34% BSC | (Iacovelli, Lanoy et al. 2012) |
| Bone metastases  (yes vs no) | NS | 16.1 vs 27.8 | 1059 | 26% | No, 4% | ND | 100% | 0% | 0% | (Motzer, Escudier et al. 2013) |
| Brain metastases  (at therapy initiation vs developed on treatment) | ND | 19.1 vs 6.3 | 705 | 0%/ND | No, 4% | ND | 72.6% | 0% | SOR 21.6%, BEV 4.7 %, TEM 0.9% | (Vickers, Al-Harbi et al. 2013) |
| Number of metastatic sites (1 vs >1) | SI (longer vs shorter), ND | SI (longer vs shorter), ND | 705 | 62% | No, 4% | ND | 0% | 0% | 0% | (Poprach, Pavlik et al. 2014) |
| Bone metastasis  (yes vs no) | ND | SI, ND | 119 | 0% | No, 6% | 111 (ND) | 69% | 0% | SOR 31% | (Zhao, Huang et al. 2014) |
| Pancreas metastasis (yes vs no) | ND | SI, ND | 119 | 0% | No, 6% | 111 (ND) | 69% | 0% | SOR 31% | (Zhao, Huang et al. 2014) |
| Response after 9 months (yes vs no) | SI, ND | SI, (longer vs shorter) ND | 38 | 26% | yes | ND | 90.5% ; | 9.5% (SOR) |  | (Dornbusch, Zacharis et al. 2013) |
| Bone metastases  (yes vs no) | ND | SI, (shorter vs longer) ND | 269 | 20% | No, 18% | ND | 82% | 0% | 18% SOR | (Park, Lee et al. 2012) |
| Liver metastases  (yes vs no) | ND | SI, (shorter vs longer ) ND | 269 | 20% | No, 18% | ND | 82% | 0% | 18% SOR | (Park, Lee et al. 2012) |
| Presence of response (yes vs no) | SI, ND | SI, ND | 1059 | 26% | No, ND (2,5% among responders)* | ND | 100% | 0% | 0% | (Molina, Lin et al. 2014) |

Legend: ND – no data, ND – Not significant (p>0.05), SI – Significant (p<0.05), SOR – sorafenib, EVE – everolimus, BSC - best supportive care, TEM – temsirolimus, BEV – bevacizumab,
